# The clinical perspective of circular RNAs in neurodegenerative diseases: potential diagnostic tools and therapeutic targets

**DOI:** 10.3389/fncel.2024.1470641

**Published:** 2024-11-18

**Authors:** Xin’ai Li, Peng Wang, Shuo Qi, Jingwei Zhou, Jeremiah Amalraj, Junhui Wang, Zhiguo Ding

**Affiliations:** ^1^Department of Thyropathy, Dongzhimen Hospital, Beijing University of Chinese Medicine, Beijing, China; ^2^The Key Laboratory of Cardiovascular Remodeling and Function Research, Chinese Ministry of Education and Chinese Ministry of Public Health, Department of Cardiology, Qilu Hospital of Shandong University, Jinan, China; ^3^Sun Simiao Institute, Beijing University of Chinese Medicine, Beijing, China; ^4^Thyropathy Hospital, Sun Simiao Hospital, Beijing University of Chinese Medicine, Beijing, China; ^5^The 1st Ward, Department of Nephrology and Endocrinology, Dongzhimen Hospital, Beijing University of Chinese Medicine, Beijing, China; ^6^Human Biology Program, University of Toronto, Toronto, ON, Canada; ^7^Lunenfeld-Tanenbaum Research Institute, Mount Sinai Hospital, Toronto, ON, Canada

**Keywords:** neurodegenerative diseases, circRNA, biomarkers, diagnosis, treatment

## Abstract

Neurodegenerative diseases (NDDs) mostly occur in older demographics. With the average lifespan increasing over time, NDDs are becoming one of the major adverse factors affecting human health and the quality of life. Currently, there are no specific diagnostic methods for NDDs and they are usually diagnosed based on nonspecific clinical symptoms and occasionally by biomarkers, such as *β*-amyloid (Aβ) for Alzheimer’s disease (AD) and a-synuclein (*α*-syn) for Parkinson’s disease, etc. However, it is usually too late for most treatment to startr when the aforementioned criteria become detectable. Circular RNAs (circRNAs) are a type of single-stranded, covalently closed, non-coding RNAs that lack a 5′ cap structure and 3′ terminal poly-A tail. According to recent research, circRNAs may play a crucial role for the onset and progression of some NDDs. These small RNAs may be potential diagnostic and prognostic markers and therapeutic targets for these diseases. This review will provide a comprehensive overview of the recent advancements of knowledge on the functions and the possible underlying mechanism of circRNAs in the pathogenesis and treatment of NDDs.

## Graphical abstract

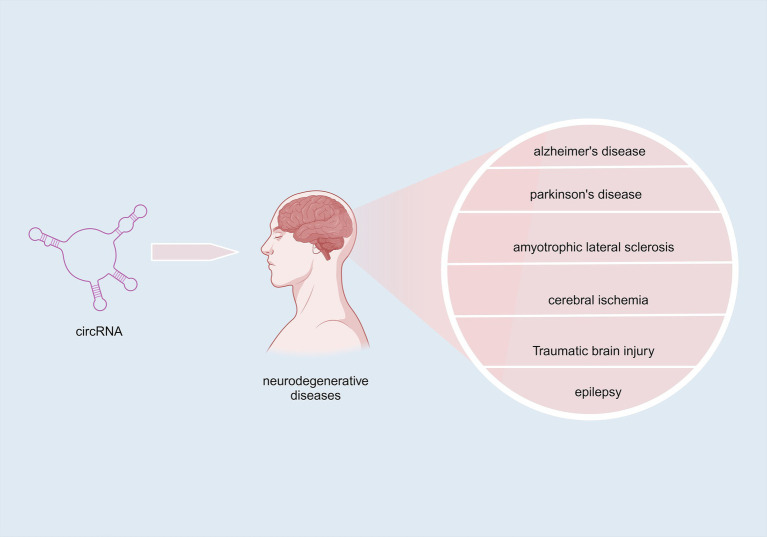

## 1 Introduction

Neurodegenerative diseases (NDDs) are a variety of brain diseases that result from the degeneration of neurons and/or their associated myelin aberrant accumulation (Dauwan et al., 2021); Jiang et al., 2023. NDDs can be categorized into acute neurodegenerative diseases, which mainly include cerebral ischemia (CI), brain injury (BI), and epilepsy, as well as chronic neurodegenerative illnesses, including but not limited to Alzheimer’s disease (AD), Parkinson’s disease (PD), Huntington’s disease, amyotrophic lateral sclerosis (ALS), spinocerebellar ataxia (SCA), and Pick disease (Xu et al., 2022; Wilson et al., 2023). Age progression, as a non- intervenable event in the life cycle, is an important predisposing factor for the development/progression of the majority of NDDs (Hou et al., 2019). Among the aging population, NDDs are now the fourth most common form of disease after cardiovascular disease, cancer, and stroke, influencing the individuals’ well-being and standard of living (Saramago and Franceschi, 2021). NDDs are characterized by complex etiology, treatment difficulty, and poor prognosis. Therefore, finding validated biomarkers of NDDs, to facilitate their diagnosis and treatment, is one of the most imperative tasks for the research community to complete (Simrén et al., 2020).

CircRNAs, which represent a type of endogenous circular noncoding RNAs (ncRNAs), were first discovered by Sanger et al. in 1976 (Sanger et al., 1976). They are generated through the splicing of exons, introns, intergenic, or untranslated regions via a non-standard splicing method called “back splicing” or “reverse splicing” (Chen, 2016; Greene et al., 2017). These specialized ncRNAs formations are controlled by spliceosomes, cis-acting components, and trans-acting variables (Ashwal-Fluss et al., 2014). They predominantly feature a stable nature, evolutionary conservatism (Li et al., 2015), and relatively high content (Ji et al., 2019; Hanan et al., 2017). As they accumulate in a number of organs, circRNAs were also demonstrated to both selectively and steadily express in the brain as well. This suggests that circRNAs may be involved in both pathological and physiological processes in the brain (Rybak-Wolf et al., 2015; Westholm et al., 2014; You et al., 2015). CircRNAs have also been implicated in a range of brain illnesses through epigenetic, transcriptional, and post-transcriptional regulation (Kristensen et al., 2019).

Compared to existing biomarkers for neurodegenerative diseases, such as Aβ and *α*-synuclein proteins, circRNAs exhibit characteristics of exceptional structural stability, tissue-specific expression, and are involved in a diverse range of mechanisms in several diseases (Kristensen et al., 2019). These features present potential advantages for the accurate diagnosis and treatment of neurodegenerative diseases associated with a given circRNA. For instance, the levels of Aβ in cerebrospinal fluid can be influenced by various factors, including age, gender, and other medical conditions. Additionally, detecting Aβ typically requires a lumbar puncture to obtain cerebrospinal fluid samples, which is an invasive procedure that many patients cannot undergo. *α*-Synuclein is primarily detected through cerebrospinal fluid or brain tissue biopsies; however, these methods also have limitations, such as the low sensitivity and specificity of cerebrospinal fluid testing, and the high-risk nature of brain tissue biopsies. In contrast, circRNA detection can be performed using less invasive procedures, such as blood tests, offering a promising new approach for diagnosing neurodegenerative diseases.

**Figure 1:**
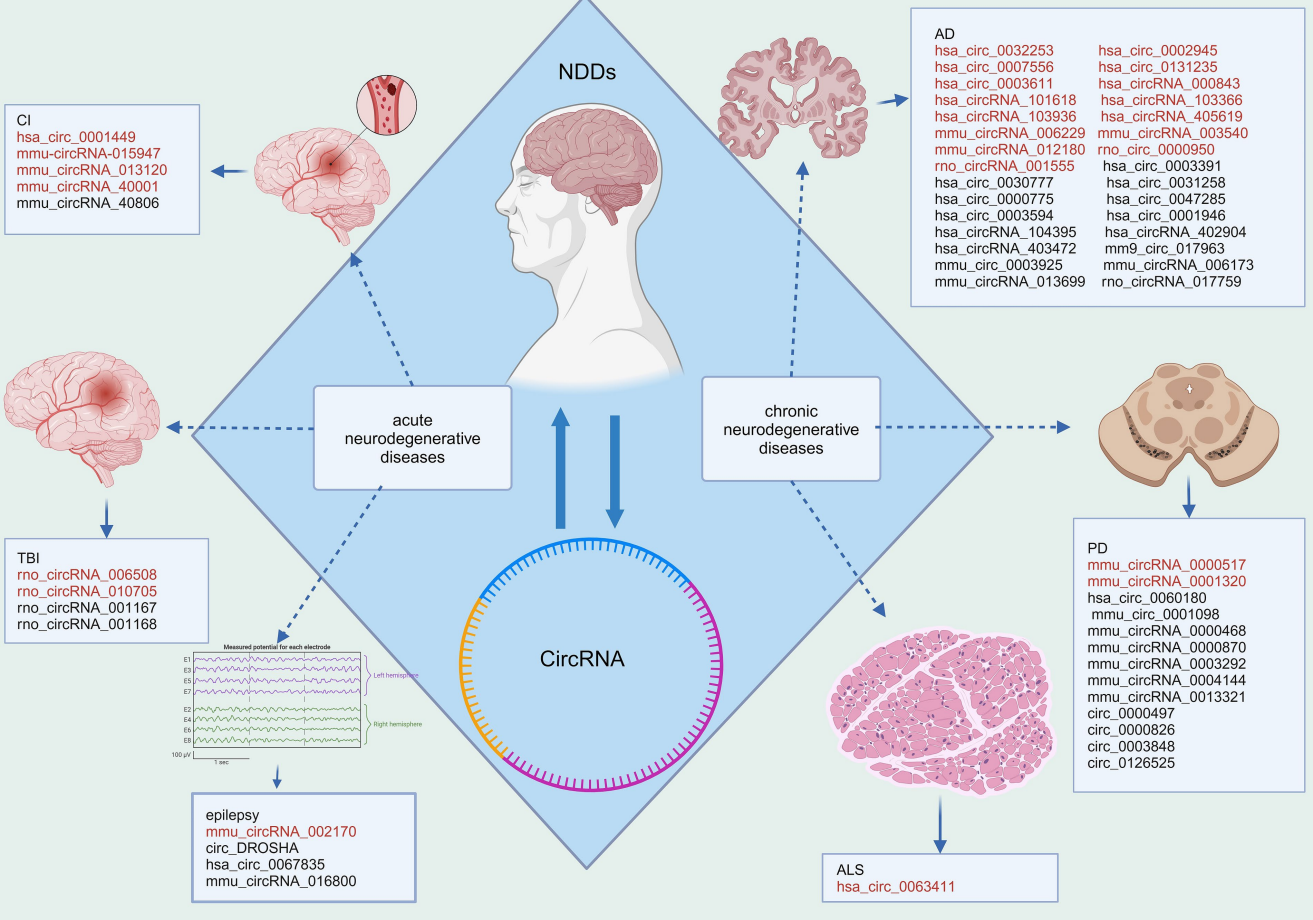
Disparities in circRNA expression in NDDs. Red fonts indicate up-regulated circRNAs in disease, while black fonts indicate down-regulated circRNAs in disease (Created with BioRender.com).

Here, we review the recent progress on the knowledge of the role of circRNA in brain diseases focusing on NDDs Figure 1 and Table 1. By summarizing these advancements in knowledge, we aim to provide guidance for researchers focusing on early diagnosis and possible novel therapies of NDDs that are applied to circRNAs.

**Table 1:** Expression and function of circRNA in NDDs.

Species	circRNA name	Host gene	Disease name	Expression pattern	Clinical significance or effect	Detect object	Detection technology	PMID
Human	hsa_circ_0001449	ARHGAP10	CI	Upregulated	Disrupting phosphatidylinositol homeostasis and AKT activity by enhancing Osbpl5 translation in transient cerebral ischemia	Tissues	RT-PCR	32,086,008
Mouse	mmu-circRNA-015947	RNF121	CI	Upregulated	Involved in OGD/ R-induced neuronal damage	Hippocampal HT22 cells	RT-PCR	26,845,359
Mouse	mmu_circRNA_013120/mm9_circ_013120	Unknown	CI	Upregulated	potential biomarkers and diagnostic tools for stroke patients	Tissues	RT-PCR	29,156,814
Mouse	mmu_circRNA_40001	Unknown	CI	Upregulated				29,156,814
Mouse	mmu_circRNA_40806	Unknown	CI	Downregulated				29,156,814
Rat	rno_circRNA_001167	Cpeb3	TBI	Downregulated	It may be involved not only in brain injury, but also in nerve regeneration after TBI	Tissues	RT-PCR	29,357,736
Rat	rno_circRNA_001168	Cpeb3	TBI	Downregulated				29,357,736
Rat	rno_circRNA_006508	LOC681740	TBI	Upregulated				29,357,736
Rat	rno_circRNA_010705	Lrp1b	TBI	Upregulated				29,357,736
Human	hsa_circ_0067835	IFT80	Epilepsy	Downregulated	Regulate the progression of refractory epilepsy by acting as a sponge for miR-155 to promote FOXO3a expression	Tissue and plasma	PCR	30,485,839
Mouse	mmu_circRNA_016800	Khdrbs3	Epilepsy	Downregulated	circRNA-miRNA-mRNA interactions regulate a variety of disease-related mrnas, thereby playing a pathophysiological role in chronic epilepsy	Tissues	PCR	30,592,747
Mouse	mmu_circRNA_002170	Unknown	Epilepsy	Upregulated				30,592,747
Human	hsa_circ_0003391	UBASH3B	AD	Downregulated	Diagnostic marker	Plasma	RT-qPCR	33,424,579
Human	hsa_circ_0030777	PCCA	AD	Downregulated	biomarker	Cerebrospinal fluid	RT-PCR	32,315,771
Human	hsa_circ_0031258	HAUS4	AD	Downregulated				32,315,771
Human	hsa_circ_0032253	GPHN	AD	Upregulated				32,315,771
Human	hsa_circ_0000775	KIF18B	AD	Downregulated				32,315,771
Human	hsa_circ_0047285	TTC39C	AD	Downregulated				32,315,771
Human	hsa_circ_0002945	AXL	AD	Upregulated				32,315,771
Human	hsa_circ_0003611	LPAR1	AD	Upregulated				32,315,771
Human	hsa_circ_0007556	Aβ-a	AD	Upregulated	The corresponding translation products may represent new therapeutic targets for AD therapy	Tissues	RT-PCR	33,003,364
Human	hsa_circ_0131235	IGF2R	AD	Upregulated	Cortical elevation of MT was significantly associated with the pathology of AD	Tissues	RT-PCR	33,704,916
Human	hsa_circ_0003594	HDAC9	AD	Downregulated	Early diagnostic markers and therapeutic targets	Serum	RT-PCR	30,887,246
Mouse	mmu_circ_0003925	Hdac9	AD	Downregulated				30,887,246
Human	hsa_circRNA_000843	Unknown	AD	Upregulated	It provides a new biomarker or therapeutic target for the control of AD	PBMC	RT-PCR	31,834,549
human	hsa_circRNA_101618	Unknown	AD	Upregulated				31,834,549
human	hsa_circRNA_103366	Unknown	AD	Upregulated				31,834,549
Human	hsa_circRNA_103936	Unknown	AD	Upregulated				31,834,549
Human	hsa_circRNA_104395	Unknown	AD	Downregulated				31,834,549
Human	hsa_circRNA_402904	Unknown	AD	Downregulated				31,834,549
Human	hsa_circRNA_403472	Unknown	AD	Downregulated				31,834,549
Human	hsa_circRNA_405619	Unknown	AD	Upregulated				31,834,549
Mouse	mm9_circ_017963	Tbc1d30	AD	Downregulated	It is helpful to understand the regulatory role of circRNAs in the pathogenesis of AD and provide valuable resources for the clinical diagnosis and treatment of AD.	Tissues	RT-PCR	29,448,241
Mouse	mmu_circRNA_006173/mm9_circ_006173	Unknown	AD	Downregulated				29,448,241
Mouse	mmu_circRNA_006229/mm9_circ_006229	Unknown	AD	Upregulated				29,448,241
Mouse	mmu_circRNA_003540/mm9_circ_003540	Usp13	AD	Upregulated				29,448,241
Mouse	mmu_circRNA_012180/mm9_circ_012180	Phkb	AD	Upregulated				29,448,241
Mouse	mmu_circRNA_013699	Unknown	AD	Downregulated				29,448,241
Rat	rno_circRNA_017759	Unknown	AD	Downregulated	Involved in the pathogenesis of AD	Tissues	RT-qPCR	29,706,607
Rat	rno_circRNA_001555	Unknown	AD	Upregulated				29,706,607
Rat	rno_circ_0000950	Unknown	AD	Upregulated	Direct sponging of miR-103 promoted neuronal apoptosis, inhibited neurite growth and increased inflammatory cytokine levels	Cells	RT-qPCR	31,373,242
Human	hsa_circ_0060180	DLGAP4	PD	Downregulated	It exerts neuroprotective effects through the miR-134-5p/CREB pathway of PD	Tissues	RT-qPCR	31,761,328
Mouse	mmu_circ_0001098	Dlgap4	PD	Downregulated				31,761,328
Mouse	mmu_circRNA_0000468	Unknown	PD	Downregulated	The mmu_circRNA_0003292-miRNA-132-Nr4a2 pathway may be involved in the regulation of the molecular mechanism of Parkinson’s disease	Tissues	RT-qPCR	32,344,560
Mouse	mmu_circRNA_0000517	Unknown	PD	Upregulated				32,344,560
Mouse	mmu_circRNA_0000870	Unknown	PD	Downregulated				32,344,560
Mouse	mmu_circRNA_0001320	Unknown	PD	Upregulated				32,344,560
Mouse	mmu_circRNA_0003292	Unknown	PD	Downregulated				32,344,560
Mouse	mmu_circRNA_0004144	Unknown	PD	Downregulated				32,344,560
Mouse	mmu_circRNA_0013321	Unknown	PD	Downregulated				32,344,560
Human	hsa_circ_0063411	TNRC6B	ALS	Upregulated	biomarker	PBMC	RT-qPCR	31,175,544

## 2 Functional mechanism of circRNAs

**Figure 2:**
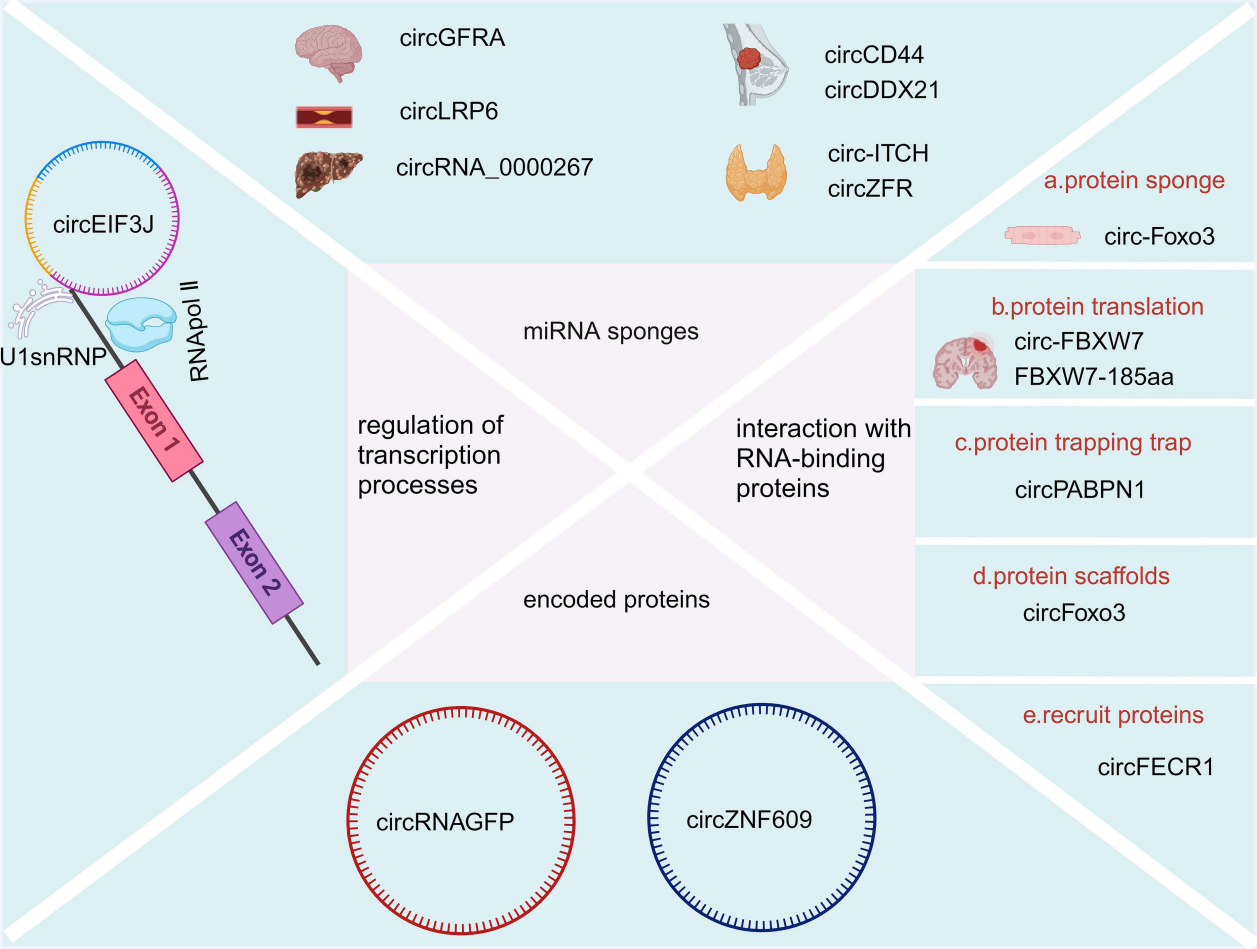
Biological function of CircRNAs (Created with BioRender.com).

Compared with linear RNAs, circRNAs lack the 5′ G-cap and 3′ -Poly-A tail structures, are not easily degraded by RNA exonucleases, and have a variety of biological property changes such as increased stability, diversity, conservatism, and specificity (Khan et al., 2022). Additionally, the half-life of circRNAs is longer than that of linear RNAs (Panda et al., 2017; Chuang et al., 2016). A variety of circRNAs, identified by deep sequencing, have been shown to have higher expression levels than their linear RNA counterparts, with some exhibiting a more than 10-fold increase (Greene et al., 2017; Wang et al., 2019). Several studies have demonstrated that circular RNAs play a significant role in the onset and progression of neurodegenerative diseases through various functional mechanisms. For instance, circRNA-7 is highly abundant in the human brain and is associated with the CircRNA of miRNA-7 (also known as ciRS-7). This molecule contains multiple tandem anti-miRNA-7 sequences, functioning as an endogenous, complement-resistant miRNA and inhibits normal miRNA activity. The inhibition of the ciR-7 “sponge effect,” elevated miRNA-7 downregulates AD-associated targets such as the ubiquitin ligase UBE2A and an autophagy-related phagocytosis protein, which is required for the clearance of amyloid peptides in the AD-affected brain (Bingol and Sheng, 2011; Lonskaya et al., 2013). Furthermore, miR-7 downregulates *α*-synuclein, thereby protecting cells from oxidative stress. Additionally, miR-7 can confer protection against 1-methyl-4-phenylpyridine (MPP+)-induced neuronal death by targeting the nuclear factor (NF)-κB signaling pathway (Shao and Chen, 2016; Choi et al., 2014). CircRNAs are known to function through several mechanisms including acting as sponges for miRNAs, interacting with RNA-binding proteins, coding for proteins, regulating transcription processes, etc. A detailed summary is illustrated in Figure 2.

### 2.1 Acting as miRNA sponges

CircRNAs consist of binding sites for microRNAs (miRNAs) and can act as sponges for miRNAs, preventing them from binding to messenger RNAs (mRNAs) and thereby influencing the expression pattern of their downstream target genes (Rong et al., 2017; Kulcheski et al., 2016). For example, silencing circGFRA1 (hsa_circ_005239) increases miR-99a expression, leading to decreased levels of p/t-AKT, p/t-FOXO1, and p/t-mTOR proteins, which eventually could suppress the migration and proliferation of glioma cells (Cao et al., 2021). Overexpression of circLRP6 contributes to atherosclerosis by sequestering miR-145 (Hall et al., 2019). Another example is Hsa_circ_0136666, which acts as a sponge for miR-136, influencing colon cancer development through the miR-136/SH2B1 axis (Jin et al., 2019). CircRNA_0000267 enhances the proliferation, differentiation, and metastasis of hepatocellular carcinoma by sequestering miR-646 (Pan et al., 2019). CircCD44 facilitates the progression of triple-negative breast cancer via its sponging action of miR-502-5p and interactions with IGF2BP2 (Li et al., 2021). CircDDX21 acts as a sponge for miR-1264, regulating QKI expression to inhibit the progression of triple-negative breast cancer (Huang et al., 2022). Overexpression of circ-ITCH sequesters miR-22-3p, leading to increased CBL expression and inhibition of the Wnt/*β*-catenin pathway, thereby hindering the progression of papillary thyroid carcinoma (Wang et al., 2018). CircZFR promotes the malignant behaviors of thyroid tumor cells via sequestering miR-1261 to upregulate C8orf4 expression (Wei et al., 2018).

### 2.2 Interaction with RNA-binding proteins

CircRNA and RNA binding proteins can interact to alter splicing patterns or mRNA stability (Holdt et al., 2016), For instance, CDR1as/ciRS-7 can bind with argonaute proteins to facilitate their degradation (Hansen et al., 2011). Additionally, circRNA has the ability to influence protein expression and function (Huang et al., 2020). For example, circ-Foxo3 can inhibit specific proteins like ID-1, E2F1, HIF-1α, and FAK, thereby preventing their nuclear translocation and inducing myocardial aging (Du et al., 2017). Du et al. also found circ-Foxo3 could bind to Foxo3 and p53 proteins. This binding shows that circ-Foxo3 facilitates MDM2-induced ubiquitination of p53 proteins and their subsequent degradation, leading to a significant decrease in p53 protein levels (Du et al., 2017). The circular RNA circSKA3 binds to Integrin beta1 proteins to induce an invadopodium formation that consequently enhances the capacity of invasion for breast cancer cells (Du et al., 2020). More interestingly, circular YAP RNAs (circYAPs) could inhibit their parental mRNA’s translation and consequently inhibit the YAP protein level in cancer cells. This sets the groundwork for exploring circYap as a possible method for intervening in cancer (Wu et al., 2019). In another study, Wu et al. found circYap was able to reduce cardiac fibrosis by binding with the proteins of tropomyosin-4 and gamma-actin, which could significantly attenuate actin polymerization in heart cells (Wu et al., 2021). Furthermore, circRNA plays a role in protein translation, with some circRNAs encoding functional proteins in human cells (Du et al., 2017). Notably, circ-FBXW7 and FBXW7-185aa have been identified as having potential prognostic significance in brain cancer (Yang et al., 2018). Another circRNA mechanism involves protein trapping, where circPABPN1 can sequester HuR, leading to the inhibition of HuR binding to PABPN1-mRNA and subsequently impeding PABPN1 translation (Abdelmohsen et al., 2017). Moreover, circRNA can act as protein scaffolds, as demonstrated by circFoxo3 (Sun et al., 2019). It also is able to recruit proteins, exemplified by circFECR1 (Chen et al., 2018).

### 2.3 Encoded proteins

Natural circRNAs have a translation start codon but no translation function, while synthetic ones can translate proteins in *in vitro* systems (Meganck et al., 2021), such as when Wang et al. discovered that circRNAGFP can translate a full-functioning GFP protein in *in vitro* cell cultures (Wang and Wang, 2015). Legnini et al. (2017) found that circZNF609 has an open reading frame with start and stop codons at both ends, is associated with a heavy chain polysaccharide, and is translated into a protein in a splice- and cap-dependent pattern. However, the efficiency of its translation was much lower than its linear mRNA counterpart.

### 2.4 Regulation of transcription processes

It has been shown that cANRIL can be formed from ANRIL transcripts after back-shearing (Burd et al., 2010). CANRIL formation reduces ANRIL and affects the binding of ANRIL to the PcG complex, which in turn affects the transcriptional regulation of INK4/ARF. In addition, circRNAPAIP2 and circEIF3J can boost the transcriptional capacities of parental genes by binding to U1 small nuclear ribonucleoprotein antibodies (U1snRNP) to form a complex, which could subsequently interact with RNA polymerase II (pol II) (Qu et al., 2015). ElciRNAs contain an intranuclear small ribonucleoprotein particle U1 (U1 snRNP) binding site, and their gene promoters can bind to U1 snRNP to form a circRNA-U1 snSNP complex and regulate gene transcription and expression through RNA polymerase II (Li et al., 2015).

## 3 Functions of circRNAs in neurodegenerative diseases

**Figure 3:**
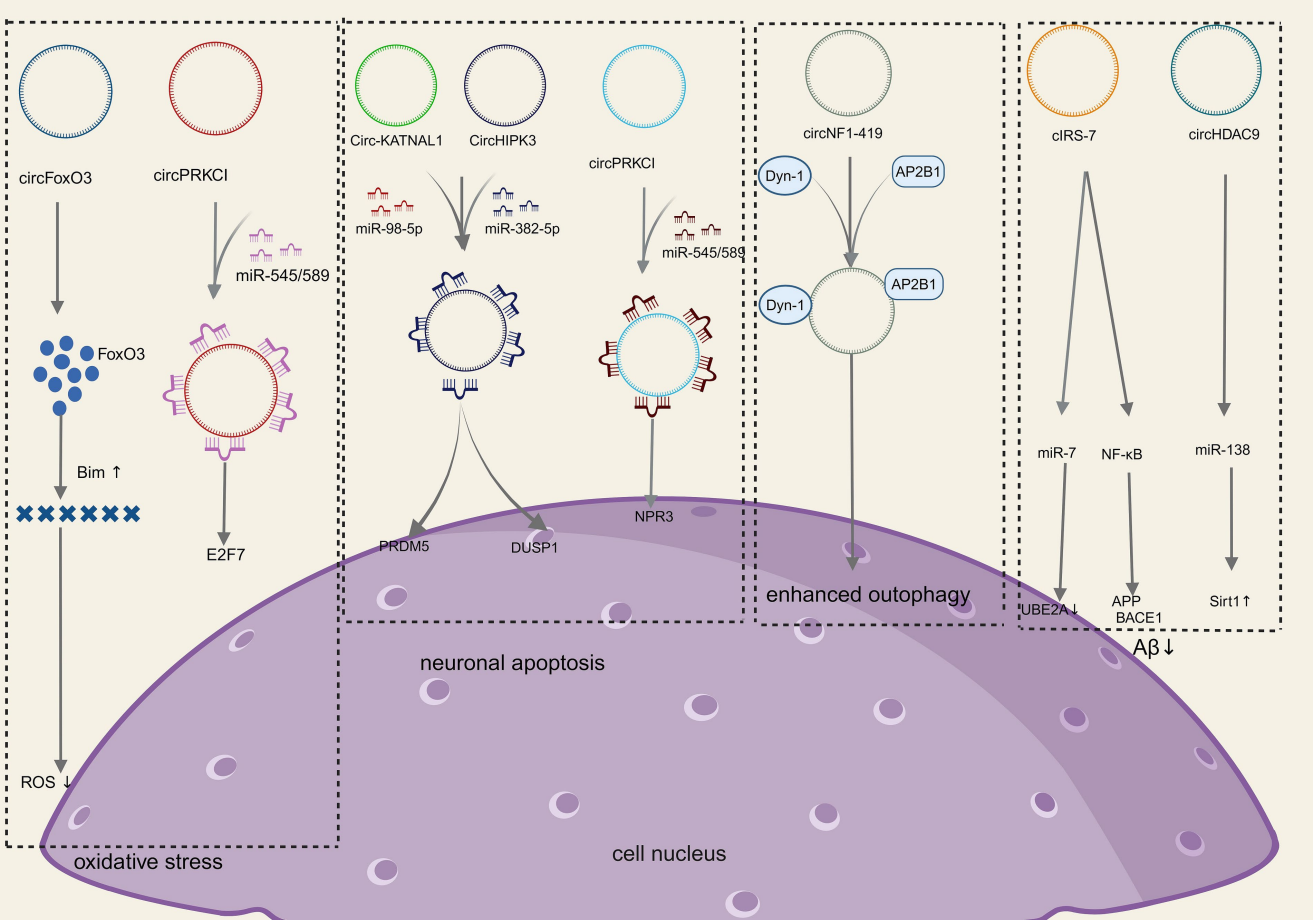
Molecular mechanism of CircRNAs in NDDs (Created with BioRender.com).

Neuronal damage and apoptosis are the most common pathological manifestations in NDDs. Oxidative stress (Singh et al., 2019), apoptosis (Gupta et al., 2021), neuronal autophagy (Pinheiro and Faustino, 2019), mitochondrial function (Johnson et al., 2021), abnormal deposition of neurotoxin proteins (Pinheiro and Faustino, 2019), immune-inflammation (Kwon and Koh, 2020), and other factors can result in neuronal damage and apoptosis as shown in Figure 3.

### 3.1 Inhibition of oxidative stress

Oxidative stress refers to the damage to nerve cells and tissues due to the excessive production or inadequate scavenging of free radicals in the nervous system and elevated levels of reactive oxygen species (ROS). Increased ROS, lipid peroxidation, and the release of cytochrome C are significant mechanisms of oxidative stress injury, which can lead to neuronal apoptosis/death and is an important basis for neurodegenerative disease progression (Cobley et al., 2018). Relevant studies have shown that the neurological damage caused by oxidative stress can be alleviated by increasing the levels of low molecular antioxidants along with antioxidant enzymes such as superoxide dismutase and glutathione peroxidase (Singh et al., 2019). Several studies have shown that circRNA is a key link between oxidative stress and neurological disease (Mehta et al., 2020). For instance, CircSLC8A1 levels rise in Parkinson’s disease and bind to Ago2 and act as sponges for miR128 targets, thereby influencing neuronal survival and aging (Hanan et al., 2020a). The endogenous circFoxo3 is crucial in initiating apoptosis and neuronal cell death. Research has shown that with the inhibition of circFoxo3 safeguarding, HT22 cells could survive oxidative harm induced by glutamate through the regulation of the mitochondrial apoptotic pathway. This suggests that circFoxo3 could be a potential therapeutic target for neurodegenerative conditions (Lin et al., 2020). Additionally, the circPRKCI-miR-545/589-E2F7 axis is involved in mediating neuronal cell damage induced by H2O2, and targeting this novel signaling pathway may offer a promising approach to protect neurons from oxidative injury as well (Cheng et al., 2019). Collectively, the aforementioned studies underscore the role of circRNA in modulating neurotoxicity induced by oxidative stress.

### 3.2 Inhibit neuronal apoptosis

Neurons survival is crucial to preserve the regular function of the nervous system. Stressors can activate intracellular signaling pathways in neurons, leading to neuronal apoptosis that results in neurological decompensation, which is the basis of neurodegenerative disease progression (Andreone et al., 2020). CircRNA_0006928 can influence neuronal apoptosis via binding to miR-184 (Zhou et al., 2017). Additionally, the overexpression of CircHIPK3 has been shown to mitigate inflammation and neuronal apoptosis in AGE1.HN cells induced by OGD through modulation of the miR-382-5p/DUSP1 axis (Yin et al., 2022). Knockdown of Circ-KATNAL1 has been found to decrease neuronal apoptosis and ameliorate spinal cord injury by acting on the miR-98-5p/PRDM5 regulatory axis (Jiang et al., 2023). Moreover, circPRDX3 can be targeted by miR-641 and miR-184 through the miR-98-5p/PRDM5 regulatory axis, influencing apoptosis in neuronal survival cells during ischemic stroke (Chen et al., 2022). Zhang et al. (2023) demonstrated that circRap1b could protect neuronal cells against apoptosis in acute ischemic stroke. Li et al. (2021) demonstrated that circPTK2 regulates sepsis-induced microglia activation and hippocampal neurons through miR-181c-5p-HMGB1 signaling, impacting activation, and hippocampal neuronal apoptosis. Zhao et al. (2020) showed that circ-HIPK3 mitigates neuronal apoptosis by regulating the miR-588/DPYSL5 axis in spinal cord injuries. Circ_0003611 has been found to regulate apoptosis and oxidative stress insults in Alzheimer’s disease (AD) through the miR-383-5p/KIF1B axis (Li et al., 2022). In Parkinson’s disease, circSAMD4A is implicated in apoptosis and autophagy in dopaminergic neurons through the miR-29c-3p-mediated AMPK/mTOR pathway (Wang et al., 2021). Circ_0000115 has been identified as a factor that prevents cerebral ischemic insults by inhibiting neuronal apoptosis, oxidative stress, and the inflammatory response through the miR-1224-5p/Nos3 axis *in vitro*, as demonstrated by Cao et al. (2024). In conclusion, all of the above studies suggest that circRNA-mediated signaling pathways play an important role in neuronal apoptosis.

### 3.3 Regulation of neuronal autophagy

Autophagy is an essential process for degrading damaged organelles and misfolded proteins in eukaryotic cells, and neurons can rely on autophagy to maintain homeostasis *in vivo*. The inappropriate accumulation of mutated and misfolded proteins inside and outside the cell is the most obvious hallmark of neurodegenerative diseases, in which the malfunction of autophagy plays a key role (Jiang and Xu, 2023). It has been shown that (Diling et al., 2019; Han et al., 2018) circRNAs slow down the progression of NDDs by influencing autophagy, which in turn reduces the buildup of misfolded proteins. In a rat sciatic nerve compression model, differentially expressed circRNAs were identified, with a downregulation observed compared to control rats. Notably, circRNA_2,837 was found to modulate neuronal autophagy by working as a sponge molecule for the miR-34 family (Zhou et al., 2018). CircRNA_2,837 knockdown can cause autophagy in spinal cord neurons *in vitro* via regulating miR-34a (Zhou et al., 2018). Additionally, circSHOC2 was discovered to inhibit neuronal apoptosis and alleviate neuronal injury by regulating autophagy through the miR-7670-3p/SIRT1 axis (Chen et al., 2020). CircEPS15 functions as a sponge for MIR24-3p, enhancing neuronal injury in Parkinson’s disease by promoting PINK1-PRKN-mediated mitochondrial autophagy (Zhou et al., 2023). CircCELF1 recruits DDX54 to upregulate NFAT5, stimulating astrocyte apoptosis and autophagy (Li and Teng, 2023). Studies have indicated that circRNAs, such as circRNA.2837 (Zhou et al., 2018) and circular RNA HIPK2 (Yang et al., 2024), play significant roles in regulating neuronal autophagy, offering potential therapeutic targets for neurodegenerative diseases. CircNF1-419 enhances autophagic activity in the cerebral cortex of SAMP8 mice by binding dynamin-1 (Dyn-1) and adapter protein 2 B1 (AP2B1) (Diling et al., 2019). In conclusion, several studies have demonstrated and concluded that circRNAs play an important role in neuronal autophagy.

### 3.4 Regulation of mitochondrial function

Mitochondria support the high demands and expenditure of the central nervous system (CNS) and are the major source of energy to maintain neuronal growth and synaptic function. Therefore, mitochondrial dysfunction is considered a key feature of neurodegenerative diseases in the CNS (Zhang et al., 2023; Lin and Beal, 2006). Mitochondrial dysfunction most likely happens in the initial stages of a variety of NDDs. When mitochondrial biogenesis is deficient, the capability of neuronal cells to clear ROS production is compromised, causing neuronal injury and apoptosis (Johnson et al., 2021). One of the primary pathological features of NDDs is the presence of misfolded and aggregated proteins in the cytoplasm, which exacerbate neuronal burden. The excessive accumulation of abnormal proteins can also harm organelles, leading to neuronal cell death and disrupting the homeostasis of the CNS (Gupta et al., 2021; Di Meco et al., 2020; Barbero-Camps et al., 2018; Nassif and Hetz, 2012). Chen et al. (2022) reported that circHIPK3 could regulate ischemic- induced apoptosis and mitochondrial dysfunction in mice by sponging miR-148b-3p via CDK5R1/SIRT1. Du et al. (2022) demonstrated that inhibition of circIgfbp2 could regulate apoptosis and mitochondrial malfunction in mice via the miR-370-3p/BACH1/HO-1 axis to ameliorate mitochondrial damage and oxidative stress-induced synaptic deficits after traumatic brain injury. Therefore, regulating mitochondrial function may offer a new therapeutic approach for NDDs.

### 3.5 Regulation of abnormal neurotoxin protein deposition

Amyloid-*β* (Aβ) protein deposition is an early pathological feature of AD (Alcendor, 2020). Soluble Aβ oligomers and Aβ protofibrils can activate glial cells, leading to the secretion of pro-inflammatory factors, which then inhibits microglia-mediated Aβ phagocytosis, ultimately resulting in Aβ aggregation and neurological damage (Pinheiro and Faustino, 2019). Studies have shown that circRNAs can actively participate in Aβ metabolism by regulating Aβ metabolism, its deposition processes, and the secretion of amyloid precursor proteins (Zhao et al., 2016; Shi et al., 2017; Lu et al., 2019; Li et al., 2020). The immune-inflammatory response generated by the brain glial cells during AD occurrence is a protective mechanism to maintain brain homeostasis. However, when microglia are repeatedly exposed to inflammation factors, they will become over-activated and release a large amount of inflammatory factors themselves, leading to neuronal death and the onset of NDDs. Since the aberrant expression of circRNAs is related to neuroinflammation as above mentioned, a series of studies have further shown that circRNAs could be potential targets for the treatment of neuro-inflammation response (Shen et al., 2019; Wang et al., 2018; Chen et al., 2019). CircRNA histone deacetylase 9 (circHDAC9) reduces Aβ production by adsorbing miR-138 and reversing the inhibition of silencing information regulatory factor-related enzyme 1 (Sirt1) by miR-138 (Lu et al., 2019). The overexpression of circHDAC9 was able to attenuate Aβ-induced neuronal damage (Zhang et al., 2020). CiRS-7 expression was decreased in the hippocampal tissues of AD patients, which led to abnormal Aβ aggregation through miR-7 (Zhao et al., 2016) and the nuclear transcription factor-κB (NF-κB) signaling pathway (Shi et al., 2017), respectively, while overexpression of CiRS-7 reduced Aβ aggregation. All of the above studies suggest that circRNA is directly or indirectly involved in Aβ aggregation.

## 4 CircRNA and chronic neurodegenerative diseases

### 4.1 CircRNA and AD

Alzheimer’s disease (AD) is a dementia disease that mostly occurs in the middle-aged and elderly population. Its characteristic pathological signs are extracellular senile plaques from β-amyloid deposition and neuronal fibrillar tangles from hyperphosphorylation of tau proteins, as well as neuronal loss with glial cell proliferation (Ma et al., 2019). It has been suggested that circRNA could play an important role in the pathogenesis of AD (Ghafouri-Fard et al., 2022). For the purpose of determining the age of circRNAs dependent dysregulation from differentially expressed circRNAs, Huang et al. employed a microarray test to study the varying expression profiles of circRNAs within the hippocampal regions of 10-month-old senescence-accelerated mouse P8 (SAMP8) and senescence-accelerated mouse drug-resistant R1 (SAMR1) mice (Huang et al., 2018). They also evaluated the distinct circRNA expression profiles among 10-month and 5-month- old SAMP8 mice. Six circRNAs were further verified by reverse transcription-quantitative polymerase chain reaction (RT-qPCR) in SAMP8 mice. qPCR results showed that hippocampal tissues of 10-month-old SAMP8, compared to age-matched SAMR1, demonstrated differentiated expression of these six circRNAs with all six circRNAs showing down-regulated expression patterns. Wang et al. (2018) performed an initial integrated microarray study on circRNA, miRNA, and mRNA expression profiles within the hippocampus of an AD rat model. Their findings also demonstrated obvious differentiated expression of certain circRNAs in AD rats compared to controls, with subsequent RT-PCR results fully supporting the findings. These results suggested that the circRNAs might interact with each other to control the expression of their target protein genes associated with the pathogenesis of AD.

By directly sponging miR-103, Yang et al. (2019) discovered that circ_0000950 induced neuronal death, suppressed neuronal synapse development, and increased the levels of inflammatory cytokines for AD. Using circRNA microarrays, Li et al. (2020) examined the circRNAs that were expressed variably within peripheral blood mononuclear cells (PBMCs) among five AD individuals and compared them with healthy controls. And their results showed that in AD patients the expressions of hsa_circRNA_000843, hsa_circRNA_101618, hsa_ circRNA_103366, hsa_circRNA_103936, and hsa_circRNA_405619 were up-regulated, whereas hsa_circRNA_104395, hsa_circRNA_402904 and hsa_circRNA_403472 expressions were down-regulated in AD patients. These results implied that circRNAs might play a role in the beginning and development of AD in humans too. Bigarré et al. (2021) discovered a significant primary factor of AD pathology with the expression of hsa_circ_0131235. Hsa_circ_0131235 had elevated expression in the temporal cortex which was substantially linked to the pathophysiology of AD.

Research focusing on circRNA as an indicator for AD diagnosis and prognosis is also becoming more popular. Lu et al. (2019) discovered that hsa_circ_0003594 was declined within the serum of individuals with mild cognitive impairment (MCI) as well as those with AD in comparison to healthy controls. Along with the down-regulation of mmu_circ_0003925 in AD mice compared to controls, the evidence suggests a potential new detectable indicator of diagnosis and a therapy target for AD. Li et al. (2020) and Li et al. (2020) evaluated circRNAs within the cerebrospinal fluid of eight AD patients, as well as eight control subjects via microarray. Ten differential expressed circRNA were further validated in the cerebrospinal fluid of another 80 AD patients and 40 controls. RT-qPCR analysis was used for the additional validation and showed that hsa_circ_0030777, hsa_circ_0031258, hsa_circ_0000775, and hsa_circ_0047285 expressions were upregulated while hsa_circ_0032253, hsa_circ_0002945, hsa_circ_0003611 expressions were down-regulated within AD patients. Their study also suggested that circ-AXL, circ-GPHN, and circ-PCCA might be useful in clinical settings for forecasting AD progression and risk. Liu et al. (2020) discovered a significant decrease in the expression of hsa_circ_0003391 in the peripheral blood mononuclear cells (PBMC) from AD patients. They also concluded that there was a correlation between the decrease and the clinical characteristics of AD patients. Their research may not only offer significant potential markers of circRNA for AD but also bring the hope of creating new AD diagnostic techniques by measuring the peripheral circRNAs. In conclusion, this research consistently validated that there are distinct expression patterns of certain circRNAs within AD patients compared to their counterparts. CircRNAs have the promising potential to become diagnostic biomarkers and therapeutic targets for AD.

### 4.2 CircRNA and Parkinson’s disease

Parkinson’s disease (PD), a progressive neurodegenerative disorder characterized by tremors and bradykinesia, is the second most common neurodegenerative disease. Its prevalence is projected to double in the next 30 years; however, accurate diagnosis of Parkinson’s disease remains a challenge and the characterization of the early stages of the disease is still ongoing (Tolosa et al., 2021; Cabreira and Massano, 2019). In general, circRNA accumulates around substantia nigra (SN) in an age-dependent manner in healthy individuals; nevertheless, this association is absent, and the overall quantity of circRNAs decreases in the SN area of PD patients. Meanwhile, some circular RNA levels were indeed elevated in some areas of the brain of individuals with PD (Hanan et al., 2020a). This suggested that circRNA might have regulatory functions in the onset and development of PD. Mutation in the SNCA gene encoding the *α*-synuclein (αSYN) protein, is one of the mechanisms that leads to PD, with αSYN expression levels abnormally elevated in the neurons of PD patients (Sun et al., 2019; Calo et al., 2016; Polymeropoulos et al., 1997). The use of dopamine agonists can down-regulate circSNCA expression levels within PD cell models, leading to miR-7 and αSYN down-regulation (Sang et al., 2018). According to other research using PD mouse and cell culture models, circDLGAP4 expression levels were down-regulated in PD, which probably indicates neuroprotective properties by affecting the miR-134-5p/CREB pathway (Feng et al., 2020). Piwecka et al. (2017) discovered that miR-7 expression was significantly reduced in the cerebellum, cortex, hippocampus, and olfactory bulb in CiRS-7 knockout mice, and that CiR-7 was reported to inhibit the expression of αSYN, suggesting that CiR-7 may play a protective role in PD. The outcomes of the research analyzing the transcriptomics of circRNAs in different brain regions of AD in a mouse model showed that mmu_circRNA_0000468, mmu_circRNA_0000870, mmu_circRNA_0003292, mmu_circRNA_0004144, mmu_circRNA_ 0013321 were down-regulated in AD and mmu_circRNA_0000517, mmu_circRNA_0001320 were up-regulated in PD. These are the results from the initial investigation of circRNA expression profiles in various brain areas in a PD animal model. The possible involvement of circRNAs within the pathophysiology of PD may be better understood in light of these findings. Furthermore, the findings suggest that the mmu_circRNA_0003292-miRNA-132-Nr4a2 pathway may be linked to the regulation of Parkinson’s disease’s molecular mechanisms (Jia et al., 2020). Additionally, some researchers have investigated circRNAs as PD biomarkers that can be used for diagnosis and prediction. PBMCs from 60 PD patients and 60 healthy control subjects were comparatively analyzed, and the microarray results indicated that the group of PD patients had considerably lower levels of six circRNAs. To further assess the diagnostic value of variously expressed circRNAs in PD patients, the area under the curve (AUC) was determined using subject workup characterization (ROC) curves and the results showed that circ_0000497, circ_0000826, circ_0003848, and circ_0126525 were significantly down-regulated within the PD group, which means they can be utilized as indicators for the detection of PD (Ravanidis et al., 2021). In conclusion, by understanding the physiological mechanisms of circRNAs, there is a clearer knowledge about their role in the pathogenesis of PD, which will give circRNAs a foundation for becoming indicators in PD diagnosis and treatment plans.

### 4.3 CircRNA and amyotrophic lateral sclerosis

As a deadly and idiopathic degenerative illness of the human locomotor system, ALS primarily causes progressive muscular atrophy, weakness, and paralysis in patients, who will eventually die from respiratory failure (Feldman et al., 2022). ALS is characterized by quick development and significant lethality, and its cause is still unclear. Functional deficiencies in ribonucleic acid processing, protein clearance, and a repetitive amplification of chromosome 9 are likely to be the underlying causes. The repeat amplification of the reading frame of 72 genes on chromosome 9 (C9orf72) is a particularly prevalent hereditary cause for ALS (Wang et al., 2021; D’Ambrosi et al., 2018; Obrador et al., 2021). There are no current clinically applicable biomarkers for ALS, and circRNAs are anticipated to serve as biomarkers for the identification of ALS and the possible development of therapeutic strategies. A study Dolinar et al. (2019) analyzed the microarray expression profiles of circRNA differences within ALS by using blood samples from 12 ALS individuals and 8 age and gender-matched normal controls. The microarray expression data were further verified by qPCR on blood samples from 60 ALS individuals and 15 age and gender-matched normal controls. Among the 10 selected circRNAs, 6 considerably up-regulated and 1 considerably down-regulated circRNAs were obtained from ALS patients, which were further analyzed by a ROC curve test. Among them, hsa_circ_0023919, hsa_circ_0088036, as well as hsa_circ_0063411 were found to be possible ALS blood indicators. The results from other studies showed an association between miR-647 expression in the spinal cord and ALS. Hsa_circ_0063411 contains a binding site for miR-647, and its parental gene TNRC6B can direct Ago-mediated gene silencing (Campos-Melo et al., 2013; Nishi et al., 2013; Dudekula et al., 2016), which may suggest that hsa_circ_006341 may be able to affect ALS by regulating miR-647. Previous studies have also shown that hsa_circ_0088036 promotes fibroblast-like synoviocytes to proliferate and migrate in RA by up-regulating SIRT1 expression and competitively binding to miR-140-3p (Zhong et al., 2020). Therefore, the aforementioned studies have indicated that circRNAs may influence the development of ALS, however, more experimental studies are still needed to identify potent circRNA markers and potential treatment targets of ALS for early diagnosis, progression prediction, and therapeutic strategies.

## 5 CircRNA and acute neurodegenerative diseases

### 5.1 CircRNA and cerebral ischemic diseases

There are currently only a few successful therapies for cerebral ischemic diseases, a disease which can cause significant brain damage, disability, and mortality. Cerebral ischemic diseases encompass cerebral ischemia–reperfusion injuries and post-stroke cerebral ischemia (Yang et al., 2018). According to Han et al., circHectd1 expression was reportedly to be substantially increased in ischemic brain areas and silencing of circHectd1 expression dramatically minimized infarct size, alleviated neuronal abnormalities, and promoted astrocyte activity (Han et al., 2018). Mechanistically, circHectd1 can function as a microRNA142 sponge that reduces miR-142 activity, which in turn suppresses astrocyte activation through macrophage/autophagy and TCDD-inducible poly (ADP-ribose) polymerase expression. Moreover, circTLK1 exacerbates post-ischemic stroke neuronal injury and neurological deficits (Wu et al., 2019). Shang et al. discovered that hsa_circ_0001449 was up-regulated within ischemic stroke areas and circ_0001449 competed alongside Osbpl5 mRNA for binding to miR-124-3p and miR-32-5p to enhance ORP5 protein levels (Shang et al., 2020). Lin et al. (2016) established the up-regulation of hsa_circ_0001449 in cultured hippocampal cells. An OGD/R (oxygen–glucose deprivation/reoxygenation) model was used in hippocampal neurons in culture, and differently expressed circRNAs in OGD/R models of cell cultures and their counterparts in cultures without ischemia were examined. 15 circRNAs were found to have significantly different expressions between the OGD/R model and the control according to circRNA microarray analysis. Utilizing qRT-PCR, the increased expression of mmu-circRNA-015947 was confirmed. Thisspecific circRNA may adsorb the miRNAs of mmu-miR-188-3p, mmu-miR-329-5p, mmu-miR-3057-3p, mmu-miR-5098, and mmu-miR-683, enhancing their target gene expression. In another study (Liu et al., 2017), temporary middle cerebral artery blockages were used to create a mouse model of focal cerebral hypoxia. CircRNA microarray analysis revealed that 1,027 circRNAs were substantially differently expressed in 48 h following ischemic cerebral reperfusion in comparison with the group that did not undergo surgery. Nineteen of them had considerably higher expression levels while the 11 of them had significantly lower levels of expression. Further qRT-PCR analysis was performed to validate the expression of three selected circRNAs and the outcomes demonstrated that the expression levels of mmu_circRNA_013120, mmu_circRNA_40001 were up-regulated, and mmu_circRNA_40806 were down-regulated. These circRNAs offer novel targets for the treatment of stroke and may also be used as biomarkers for indicating the progression of cerebral ischemia. In a focal cerebral ischemia–reperfusion mouse model, circUCK2 decreased oxygen and glucose deprivation (OGD)-induced apoptosis by controlling transforming growth factor *β* (TGF-β)/Smad3 signaling (Chen et al., 2020). The up-regulation of circUCK2 levels dramatically decreased infarct size, mitigated neuronal injury, and improved neurological impairment. Furthermore, circUCK2 functioned as an endogenous sponge for miR-125b-5p and stifled its action, resulting in elevated expression of growth differentiating factor and a consequent reduction in neuronal damage. The above research has suggested that circRNAs could be biomarkers of cerebral ischemia injury progression and might be therapeutic targets for treatment. However, these studies are preclinical studies, and clinical trials are warranted to validate this claim before they can be used in clinical applications.

### 5.2 CircRNA and traumatic brain injury

TBI is a brain injury that results from mechanical external forces, causing either temporary or permanent damage (Capizzi et al., 2020) and constitutes one of the primary contributors of chronic disability and mortality globally. There is no evidence to suggest that current pharmacologic interventions can enhance outcomes following TBI (Cancelliere et al., 2017; Brazinova et al., 2021; El-Menyar et al., 2017). A full set of 192 circRNAs was identified to be differently expressed in TBI rats according to a study that used microarrays to show changed circRNAs in the hippocampal regions of the rats. They identified 98 up-regulated and 94 down-regulated circRNAs. Both GO and KEGG pathways analysis indicated that mRNAs transcribed from multiple differentially expressed circRNA host genes were associated with brain injury and neurodegeneration. Furthermore, RT-PCR was employed to confirm the distinct expression of four circRNAs and the results showed that the expression of rno_circRNA_001167 and rno_circRNA_001168 were down-regulated, while the expression of rno_circRNA_006508 and rno_circRNA_010705 were up-regulated. These results revealed circRNA role in functional dysregulation within the pathophysiological process after TBI. Nevertheless, additional investigation from both preclinical and clinical settings are necessary to further understand the biological roles of these circRNAs (Xie et al., 2018).

### 5.3 CircRNA and epilepsy

Epilepsy is one of the most prevalent neurological disorders, with the highest risk of development occurring in infants and the elderly (Thijs et al., 2019; Manford, 2017). Epilepsy is a clinical phenomenon caused by highly coordinated aberrant neuronal discharges in the brain (Beghi et al., 2015). NcRNAs are involved within the control of pathological and physiological activities of the brain and are dysregulated during epileptogenesis (Shao and Chen, 2017). CircRNAs may be crucial in the onset of epilepsy and may serve as biomolecular targets and markers for clinical diagnosis. Gong et al. (2018) found that an overall of 586 differently expressed circRNAs were identified in temporal lobe epilepsy (TLE) compared to control tissues. CircRNA-0067835 expression was notably down-regulated within TLE patients’ plasma and tissue samples. Lower circRNA-0067835 was significantly associated with higher seizure frequency, hippocampal sclerosis, and higher Engel scores. Utilizing a luciferase reporter gene test, circRNA-0067835 was found to function as a miR-155 sponge to affect FOXO3a expression. Research has demonstrated that circRNA-0067835 serves to be a miR-155 sponge to enhance the expression of FOXO3a, hence controlling the development of refractory epilepsy. This finding raises the possibility that circRNA-0067835 could be a therapeutic target for individuals with TLE. 17 TLE and 17 non-TLE patients had their temporal lobe cortex obtained by Li et al. (2018). Utilizing high-throughput sequencing, total RNA was extracted and the transcriptome of dysregulated circRNAs was examined. Quantitative PCR was performed to validate the altered circRNAs. 442 circRNAs were found to be expressed differently within the TLE and non-TLE controls, among them, 188 circRNAs were up-regulated and 254 were down-regulated in the TLE patients. With quantitative PCR, 8 circRNAs were further verified. Notably, circ-EFCAB2 was strongly upregulated in the TLE category, whereas the expression of circ-DROSHA was considerably reduced in the non-TLE category. Bioinformatics analysis revealed that the binding of circ-EFCAB2 to miR-485-5p increased the expression level of the ion channel CLCN6, whereas the interaction of circ-DROSHA with miR-1252-5p decreased the expression levels of ATP1A2. The pathophysiology of TLE may be reflected in the dysregulation of circRNAs. And circ-EFCAB2 and circ-DROSHA may represent viable targets for therapy. Zheng et al. (2021) showed that circ_DROSHA was downregulated in the samples of TLE patients’ serum. In a TLE cell model, they found that circ_DROSHA regulates MEF2C expression via competitive binding to miR-106b-5p and affects cell injury in TLE cell models. CircUBQLN1 prevents neurological injury in Mg^2+^-treated human hippocampal neuron (HN-h) cells by controlling the miR-155/SOX7 axis, which suggests that circUBQLN1 could be employed as a treatment target during epilepsy therapy (Zhu et al., 2021). Lee et al. (2018) discovered that 43 circRNAs were dysregulated in the hippocampus of an epilepsy mouse model, among which 26 were up-regulated and 17 were down-regulated. Changes in miRNA response elements (MRE) expression in these circRNAs were inversely connected to shifts in the target miRNAs’ expression, supporting the claim that circRNAs could inhibit the expression of their target miRNAs. Further PCR validation showed that mmu_circRNA_016800 expression was down-regulated, while mmu_circRNA_002170 expression was up-regulated, suggesting that dysregulated circRNAs could regulate multiple disease-associated mRNAs through circRNA-miRNA-mRNA interactions, thereby contributing to the pathophysiology of persistent epilepsy.

## 6 Potential applications of CircRNAs in NDDs

Firstly, circRNAs can serve as diagnostic markers for NDDs (Chen, 2016; Kristensen et al., 2019; Verduci et al., 2021). CircRNAs are structurally stable and hold significant potential for the early diagnosis and monitoring of NDDs. The detection of specific circRNAs in bodily fluids, such as blood and cerebrospinal fluid, can be utilized as a biomarker of disease (Beylerli et al., 2024). Some circRNAs are uniquely expressed in specific nerve cell types or brains, and thus can be used as diagnostic markers for NDDs. Secondly, circRNAs can be used as therapeutic targets for NDDs. CircRNAs can regulate gene expression through multiple mechanisms, thus affecting the development of NDDs. circRNAs can regulate gene expression, modulate neuroinflammation, affect protein aggregation, interact with proteins, and influence protein function and activity, providing new targets for the treatment of NDDs (Sang et al., 2018; Titze-de-Almeida and Titze-de-Almeida, 2023; D'Anca et al., 2022). Thirdly, it can act in drug development and delivery. Circular RNA itself can be used as a target for drug development. By screening small molecule drugs or antibodies against specific circRNAs, the expression or function of circRNAs can be regulated to treat NDDs. In addition, circRNAs have the characteristics of low immunogenicity and high stability, which makes them a potential carrier for drug delivery. Drugs for the treatment of neurodegenerative diseases can be encapsulated in circRNA and delivered to specific nerve cells or brain regions through this carrier, improving the therapeutic effect of the drugs while reducing the toxic side effects of drugs carriers on normal tissues (Feng et al., 2020; Bai et al., 2018; Liu et al., 2022; Cheng et al., 2022; Hanan et al., 2020b).

### 6.1 Limitations and future outlook

The incidence of non-communicable diseases, such as NDDs and other geriatric illnesses has increased as a result of population aging (Gitler et al., 2017). The complex pathogenesis of these diseases, the insidious nature of its early changes, and the lack of diagnosis and treatment-related markers contribute to some of the characteristics of NDD treatment, such as difficult early diagnosis, lack of efficient treatments, and poor prognosis. CircRNAs are involved in a range of events in cells, which include cell proliferation, motility, immunity, apoptosis, senescence, and oxidative stress (Misir et al., 2022). CircRNAs’ unique structural composition makes them highly distinctive players that are different from other cell molecules and also allows them to produce tissue-specific expression patterns (Misir et al., 2022). Many circRNAs are stable and express themselves specifically in terms of tissues or organs, including the brain, which makes them potential good candidates for indicators of CNS diseases such as NDDs.

However, there are still some limitations in the current study of circRNAs in NDDs. Firstly, most of the differentially expressed circRNAs identified through microarray or RNA sequencing analyses have not yet been validated in human samples, and although some circRNAs have been preliminarily validated, the analyses utilized small sample sizes, which may reduce the statistical efficacy. Secondly, most mechanistic studies of NDDs have been confined to *in vitro* cellular studies, and some *in vivo* level studies are lacking. Thirdly, current studies primarily focus on blood specimens, while data from other body fluids and lesion specimen studies are still lacking and need to be supplemented. Fourthly, current studies evaluate the associations between dysregulated circRNAs, microRNAs (miRNAs), and mRNAs, and further validation of the regulatory interactions among them is still needed. Fifthly, the specific molecules and pathways upstream and downstream of circRNAs are not yet fully understood. Based on this, more research on the role of circRNAs in NDDs and the healthy brain is needed, and it is recommended that all types of regulatory RNAs and mRNAs be included in these studies and that more advanced circRNA data analysis and bioinformatics algorithms be used to comprehensively understand the interactions of circRNAs in NDDs. This is of great scientific significance for the in-depth understanding of the mechanism of NDDs, and more importantly, for the clinical development of NDDs through potential targets and means for clinical diagnosis and treatment of NDDs. It is expected that the rapid development of biotechnology and clinical research will promote the role of circRNAs in the diagnosis and treatment of NDDs.
